# Maintaining periodontally compromised teeth seems more cost-effective than replacing them with dental implants

**DOI:** 10.1038/s41432-024-01050-2

**Published:** 2024-08-19

**Authors:** Kelvin I. Afrashtehfar, Nasser M. Assery, Khaled A. K. Alblooshi, Patrick R. Schmidlin

**Affiliations:** 1 Department of Reconstructive Dentistry and Gerodontology, School of Dental Medicine, University of Bern, Bern, Switzerland; 2 College of Dentistry, Ajman University, Ajman City, UAE; 3 Dental Consultant, Private Practice, Abu Dhabi City, UAE; 4 Department of Periodontology, Eastman Institute for Oral Health, Rochester, University of Rochester, Rochester, NY USA; 5 Division of Periodontology and Peri-implant Diseases, Clinic of Conservative and Preventive Dentistry, Center of Dental Medicine, University of Zurich, Zurich, Switzerland

**Keywords:** Periodontics, Dental implants

## Abstract

**Design:**

A systematic appraisal without statistical aggregation.

**Data sources:**

The researchers utilized Ovid (Medline), Embase, Web of Science, and CINAHL databases. They performed a comprehensive literature search, which concluded in July 2023. References of selected studies and systematic reviews were examined for additional relevant articles.

**Study selection:**

The review included studies (randomized controlled trials [RCTs], systematic reviews [SRs], narrative reviews, retrospective studies, cross-sectional studies, case series, case reports) discussing the cost-effectiveness of preserving teeth versus replacing them with implants in patients with severe periodontal disease. Non-English publications, letters, conference abstracts, and brief reports were excluded.

**Data extraction and synthesis:**

Two reviewers independently screened titles and abstracts using a systematic review screening tool, resolving conflicts with a third reviewer. The extracted data included study design, patient demographics, treatment details, economic models, costs, and clinical implications. Quality was assessed using Joanna Briggs Institute (JBI) critical appraisal tools, with scores converted to percentages.

**Results:**

A total of 633 studies were identified for periodontal treatment, with 9 included after screening, while 114 studies were identified for dental implants, of which 3 were included after screening. The included studies were published between 2008 and 2018, predominantly from Germany, and mainly comprised retrospective designs, along with some prospective and model-based analyses. Follow-up periods ranged from 36 months to 33 years. Treatment costs were found to increase with disease severity, with surgical treatments being more expensive than nonsurgical ones, and supportive periodontal treatment (SPT) representing the highest cost share in periodontal treatment. Maintaining implants proved more costly than maintaining teeth, especially in cases of peri-implantitis. For chronic periodontitis, total treatment costs per tooth were €222 ± €98 over 18.7 years, and for aggressive periodontitis, €267 ± €148 over 16.9 years. Regular SPT cost €806 per tooth per year over 28.7 years, with significant cost variations across studies. A 2013 study found that maintaining implants was five times costlier than maintaining teeth, particularly if peri-implantitis developed. A 2018 study indicated that implant-supported crowns (ISCs) were the most expensive therapy. Only one study directly compared costs within the same patient, finding periodontal treatment to be more cost-effective than implants. Costs also increased with irregular SPT, aggressive periodontitis, and specialist treatments compared to regular SPT, chronic periodontitis, and treatments by general dental practitioners. The quality of included studies scored between 45% and 84.6%, indicating moderate to high quality, with methodological issues including unclear strategies for handling confounding factors and incomplete follow-up.

**Conclusions:**

Implants are effective for replacing missing teeth but are associated with higher long-term costs and complications. Maintaining periodontally compromised teeth is generally more cost-effective, therefore, maintenance costs and potential complications should be carefully considered in treatment planning. There is a need for studies comparing the long-term cost-effectiveness of saving teeth compared to replacing them with implants, considering several variables for informed clinical decision-making.

## A Commentary on

**Nagpal D, Ibraimova L, Ohinmaa A, Levin L**.

The cost-effectiveness of tooth preservation vs implant placement in severe periodontal disease patients: a systematic review. *Quintessence Int*. 2024; 10.3290/j.qi.b4500025.

**GRADE Rating:** 

## Commentary

Severe periodontitis affects approximately 11% of the global population, as indicated by the Global Burden of Disease Study 2017 and the European Federation of Periodontology (EFP)^1^. This condition leads to the loss of periodontal support, impairing chewing ability and negatively impacting oral health-related quality of life^2^. Treatment success centers on several factors including home care, dental hygiene, risk management, disease control, and supportive periodontal therapy (SPT)^3^. Despite its chronic nature, regular SPT is required to maintain long-term stability^4^. Modern dental implants are popular for replacing missing teeth, but accurate diagnosis and prognosis assessment are necessary before deciding on treatment^5^. Despite high survival rates, implants can fail, especially with peri-implant inflammations like mucositis and peri-implantitis^6,7^. Levin and Halperin-Sternfeld found implant survival does not exceed that of properly maintained natural teeth^8^. Thus, economic evaluations are fundamental for deciding treatment strategies, considering costs and outcomes, making it an area requiring further investigation. The reviewed meta-analysis by Nagpal et al.^9^ aimed to determine the cost-effectiveness of periodontal treatment versus dental implants for periodontally compromised teeth, considering both clinical and economic aspects.

The primary strength of this systematic review is its adherence to the PRISMA statement guidelines, providing a robust and transparent methodological framework. The comprehensive search strategy across 4 databases up to summer 2023 supported the thoroughness and reliability of the review. Additionally, the inclusion criteria were well-defined, including various study designs (RCTs, SRs, narrative reviews, retrospective studies, cross-sectional studies, case series, and case reports) which allowed for a broad assessment of the available evidence.

The independent screening and quality assessment by two reviewers, with a third reviewer resolving conflicts, minimized bias and confirmed the reliability of the selection process. The use of the JBI critical appraisal tools for evaluating the methodological quality of included studies further strengthened the review’s findings^10^. The detailed data extraction process, capturing key aspects such as study design, patient demographics, treatment modalities, and economic models, provided a comprehensive overview of the studies’ characteristics and outcomes.

A significant limitation of this review is the heterogeneity among the included studies, which did not permit conducting a meta-analysis. This variability in study designs, populations, and follow-up durations complicates direct comparisons and generalizations of the findings. Additionally, the review predominantly included retrospective studies, which may be prone to selection and recall bias. The lack of randomized clinical trials with long-term follow-up also limits the strength of the evidence.

Future research should conduct robust RCTs with long-term follow-up to provide clear evidence on the cost-effectiveness of preserving teeth versus dental implants in severe periodontal disease. Standardized methodologies and consistent outcome definitions are needed for reliable comparisons. Additionally, economic evaluations should include broader costs and patient-reported quality of life and functional outcomes^11,12^.

To sum up, while the current review determined the cost-effectiveness of periodontal treatment versus dental implants, the findings are limited by the heterogeneity and predominance of retrospective studies. Despite these limitations, the review identified the potential for periodontal treatment to be a cost-effective alternative to dental implants, particularly when considering long-term maintenance and complication costs. Thus, future research should aim to address the identified limitations and provide more robust evidence to inform clinical decision-making.

Practice points

- Maintaining periodontally compromised teeth is generally more cost-effective than replacing them with dental implants, considering long-term maintenance costs.
- Dental implants, although effective, require strict maintenance and present potential complications that can increase overall treatment costs.
